# Effects of emotion regulation and perpetrator-victim roles in intimate partner violence on mental health problems among men who have sex with men in China

**DOI:** 10.1017/S2045796020000712

**Published:** 2020-08-14

**Authors:** D. Wei, F. Hou, W. Cao, C. Hao, J. Gu, L. Peng, J. Li

**Affiliations:** 1 School of Public Health, Sun Yat-Sen University, No.74, Zhongshan second road, Guangzhou, China; 2 Department of Public Mental Health, Shenzhen Kangning Hospital, No. 1080 Cuizhu Road, Luohu District, Shenzhen, Guangdong 518020, China; 3 Sun Yat-sen Global Health Institute, Sun Yat-sen University, Guangzhou, China; 4 Department of Health Services, Policy and Practice, School of Public Health, Brown University, Providence, Rhode Island, USA

**Keywords:** Emotion regulation, intimate partner violence, mental health, men who have sex with men, perpetrator-victim role

## Abstract

**Aims:**

This study assessed the relationships between different perpetrator-victim roles in intimate partner violence (IPV), emotion regulation (ER) and mental health problems among men who have sex with men (MSM) in China.

**Methods:**

From April to June 2019, 1233 participants were approached via gay-friendly non-governmental organisations in 15 cities across mainland China.

**Results:**

Of the total, 578 eligible participants completed an anonymous online survey. All participants provided informed consent and information about their violent perpetrator-victim role and mental health status. The results revealed a high prevalence of IPV in this study sample, with 32.7% of participants reporting IPV victimisation and 32.5% of participants reporting IPV perpetration during their lifetime. A total of 81 (14.0%) participants were suicidal, 309 (53.5%) participants reported poor general mental health and 208 (36.0%) had significant depressive symptoms. Adjusted logistic regression models revealed that both physical victimisation (adjusted odds ratio [ORa] = 3.22, 95% confidence interval [CI] = 1.11–9.32) and sexual victimisation (ORa = 2.90, 95% CI = 1.39–6.05) had positive associations with suicidality, and unidirectional and bidirectional psychological perpetration were associated with poor general mental health and significant depressive symptoms. Although high cognitive reappraisal showed a negative association with poor general mental health (ORa = 0.89，95% CI = 0.86–0.92), the correlation with victims of IPV was weaker than it was with non-victims.

**Conclusions:**

This study revealed that different perpetrator-victim roles in different IPV situations should be considered comprehensively in research, prevention and intervention. ER is not enough to buffer the effects of IPV on the mental health of MSM victims.

## Introduction

According to limited studies, Chinese men who have sex with men (MSM) have a high prevalence of intimate partner violence (IPV) (Dunkle *et al*., 2013; Davis *et al*., 2015; Liu *et al*., 2018; Wang *et al*., 2018). These studies have also reported an association between IPV and high-risk sexual behaviour or human immunodeficiency virus (HIV) infection. In addition, the positive association between IPV and adverse mental health outcomes (i.e. substance abuse and depression) in the MSM population has been well documented abroad (Buller *et al*., 2014). However, few studies have focused on the association between mental health problems and IPV among MSM in China.

There is ample evidence that MSM are disproportionately vulnerable to various psychological problems, including depression, anxiety, substance abuse and suicidality (O'Cleirigh *et al*., 2015; Batchelder *et al*., 2017). Research abroad has revealed that the association between IPV and mental health outcomes varies with different perpetrator-victim roles (Graham *et al*., 2012; Ulloa and Hammett, 2016). It has been suggested that MSM who are victims of IPV are more likely to suffer from depressive symptoms and suicidality (Devries *et al*., 2013; Buller *et al*., 2014; Parker *et al*., 2015; Kazan *et al*., 2016), and the prevalence and severity of mental health problems are higher among individuals who are both perpetrator and victim (bidirectional violence; Ulloa and Hammett, 2016). However, few studies have measured IPV perpetration and its effect on mental health in China. Even fewer studies have examined the emergence of bidirectional violence and explored its effects on mental health (Buller *et al*., 2014). The association between different experiences of IPV (e.g. victim *v*. perpetrator, physical violence *v*. psychological violence) and mental health problems other than depression has also been rarely studied.

However, the role of emotion regulation (ER) has attracted increasing attention in IPV and mental health research. Studies have shown that men with high ER difficulties tend to show aggressive behaviour physically and sexually (Shorey *et al*., 2015; Kirwan *et al*., 2019). In addition, emotional disorders can lead to a risky-taking personality and risky characteristics for a multitude of personal, emotional and mental health problems (Gross, 2002). Two main ER strategies have been widely studied: cognitive reappraisal and expressive suppression (Gross and John, 1998). Cognitive reappraisal is a form of cognitive change that involves construing a potentially emotion-eliciting situation in a way that changes its emotional effect (Lazarus and Alfert, 1964), and expressive suppression is a form of response modulation that involves inhibiting ongoing emotion-expressive behaviour (Gross and John, 1998). Studies have shown that the use of cognitive reappraisal is associated with healthier affect patterns (Cutuli, 2014) and that the use of suppression usually leads to adverse effects (Gross and John, 2003). The meta-analysis conducted by Hu *et al*. (2014) also revealed that cognitive reappraisal is associated with better mental health by increasing life satisfaction and positive effects, whilst expressive suppression does the opposite.

In this context, we hypothesise that ER can moderate the association between IPV and mental health, in which cognitive reappraisal acts as a buffer against the adverse effect of IPV, whilst expressive suppression is a vulnerability factor that leads to mental health problems. The objectives of this study were (1) to explore the prevalence of different perpetrator-victim roles in different types of IPV; (2) to assess the relationships between different IPV experiences, ER and mental health problems, including suicidality, general mental health and depressive symptoms; and (3) to test the hypothesis that ER can moderate the association between IPV and mental health problems.

## Methods

### Participants and procedure

This cross-sectional study surveyed MSM in 15 cities across China, including five in eastern China (Sanya, Fuzhou, Hangzhou, Shenzhen and Qingdao), three in central China (Taiyuan, Changsha and Hefei), three in northeast China (Changchun, Zhengzhou and Harbin) and four in western China (Lanzhou, Nanning, Urumqi and Kunming). The participants were recruited via gay-friendly non-governmental organisations in local cities between April and June 2019. They were invited to complete a self-administered online survey, which stated that participation was anonymous and voluntary, that declining to participate would have no consequences and that the data would only be used for research purposes. The inclusion criteria were (1) age of at least 18 years, (2) men self-reported anal intercourse with at least one man in the last 6 months and (3) one or more intimate partners. In this study, ‘intimate partner’ was defined as a primary male partner that a participant was dating or with whom a participant had an ongoing intimate relationship (Davis *et al*., 2016). The questionnaire took about 20 min to complete, and the participants received monetary compensation of RMB15 (about US$2.5) for their time. In total, 1233 eligible participants were approached, of whom 578 completed the online survey and were included in the final study (response rate, 46.9%).

We obtained ethical approval from the Ethics Committee of Sun Yat-Sen University ([2018] 049). In this study, we asked all participants to give their informed consent before completing the online survey, which specifically informed them that the research team would contact them if their responses revealed any suicidal ideation, suicide plans or suicide attempts. For suicidality, team members in local non-governmental organisations would call the participants, inform them about crisis intervention services and help them use these services if necessary.

### Measures

#### Demographics

Socio-demographic information was collected, including age, city of residence, ethnicity, education level, monthly income, employment status, marital status and sexual orientation.

#### IPV

This study used five questions to capture five types of IPV. The specific acts described in these types of IPV were derived from the intimate partner violence among gay and bisexual men (IPV-GBM) scale, specifically developed to assess the presence of IPV among MSM (Stephenson and Finneran, 2013). This scale has excellent internal reliability (Cronbach's Alpha > 0.90) and has been used in several studies (Stephenson *et al*., 2013; 2016; Stephenson and Finneran, 2017; Wei *et al*., 2019). To measure different perpetrator-victim roles in IPV (i.e., non-violence, perpetrator-only, victim-only and bidirectional violence), the answers to each question were classified into four categories: (A) I did the above to my partner; (B) My partner did the above to me; (C) Both A and B; and (D) Neither A nor B. A, B, C and D thus represented perpetrator-only, victim-only, bidirectional violence and non-violence, respectively. In addition, the participants who chose A or C were considered IPV perpetrators, and those who chose B or C were recorded as IPV victims. The Cronbach's alpha value for this scale was 0.71 in this study.

#### Suicidality

Suicidality covers suicidal ideation, suicide plans and suicide attempts. The participants were asked to respond to three items on their suicidality. The questions were, ‘Have you had suicidal thoughts in the past 3 months?’, ‘Have you specifically planned to commit suicide in the past 3 months?’ and ‘Have you attempted suicide in the past 3 months?’ For each item, the participants answered no (0) or yes (1). Those who answered ‘yes’ to any of the three questions were recorded as positive for suicidality (Ulloa and Hammett, 2016).

#### General mental health

General mental health status was measured with the 12-item general health questionnaire (GHQ-12; Goldberg and Williams, 1988). This study used the GHQ scoring method (0-0-1-1) rather than that of a simple Likert scale (0-1-2-3) because this method can help eliminate any bias from participants who tend to choose answers 1 and 4 or 2 and 3, respectively (Goldberg and Williams, 1988). Based on the recommendations of previous studies (Goldberg *et al*., 1998), the mean GHQ score (3.1) of this sample was used as a rough indicator for the cut-off point, and those who with a score greater than 3 were considered to have poor general mental health. The Cronbach's alpha value for this scale was 0.78 in this study.

#### Depression

A 10-item version of the Centre of Epidemiological Studies Depression Scale (CES-D) was used to confirm whether the participants had significant symptoms of depression (Radloff, 1977). All items were added to obtain the total scores, with a higher score indicating more severe depression. A cut-off score of 10 or higher indicated the presence of significant depressive symptoms (Miller *et al*., 2008; Zhang *et al*., 2012). The Cronbach's alpha value for this scale was 0.89 in this study.

#### Emotion regulation

ER was measured by the Emotion Regulation Questionnaire (ERQ) consisting of 10 items and divided into two independent subscales (Gross and John, 2003). Six items were used to measure the participants' cognitive reappraisal (i.e. the ability to control their emotions by changing the way they think about their situation) and four items were adopted to measure expressive suppression (i.e. the ability to control their emotions by not expressing them). All items were rated on a 7-point Likert scale, ranging from strongly disagree (0) to strongly agree (6), with a higher score indicating more frequent use of ER strategies. In this sample, the ERQ had a good internal consistency with Cronbach's alpha value of 0.89 and 0.78 for cognitive reappraisal and expressive suppression, respectively.

### Statistical analysis

Multiple logistic regression models were fit for the three dependent variables (i.e., suicidality, poor general mental health and significant depressive symptoms), adjusted for background variables that were significant or marginally significant in univariate analysis (*p* < 0.1). The adjusted odds ratios (ORa) and their 95% confidence interval (CI) were obtained. In addition, Spearman's nonparametric rho correlations were used to show the inter-correlations between the two independent variables (IPV victimisation and IPV perpetration), the two moderators (cognitive reappraisal and expressive suppression) and the three mental health outcomes (suicidality, poor general mental health and significant depressive symptoms). Hierarchical logistic regression was conducted to examine the moderating effect of ER on IPV victimisation and IPV perpetration. The variables were added in four blocks: (1) background covariates; (2) IPV victimisation or IPV perpetration; (3) ER subscales (i.e., cognitive reappraisal and expressive suppression); and (4) interaction terms (i.e., IPV victimisation × cognitive reappraisal, IPV victimisation × expressive suppression, IPV perpetration × cognitive reappraisal, IPV × expressive suppression). A *p* value of 0.05 was set as the level of statistical significance. All analyses were conducted in SPSS version 25.

## Results

### Descriptive statistics

Most participants (62.8%) were younger than 30 years. More than half had completed university education (54.3%) and worked full-time (66.1%). In addition, most were homosexual (81.3%) and unmarried (51.2% were single and 36.0% had a boyfriend[s]). Details are listed in Table 1. Overall, we found no socio-demographic difference between the perpetrators and the non-perpetrators or between the victims and the non-victims (*p* < 0.05).

**Table 1.** Socio-demographic Characteristics of sample MSM population

	Any IPV perpetration *n* (%)			Any IPV victimisation *n* (%)			Total *N* (%)
	No	Yes	*p*	No	Yes	*p*	
Age							
<30	240 (41.5)	123 (21.3)	1.00	238 (41.2)	125 (21.6)	1.00	363 (62.8)
⩾30	150 (26.0)	65 (11.2)	0.37	151 (26.1)	64 (11.1)	0.25	215 (37.2)
Ethnicity							
Others	33 (5.7)	17 (2.9)	1.00	30 (5.2)	20 (3.5)	1.00	50 (8.7)
Han	357 (61.8)	171 (29.6)	0.93	359 (62.1)	169 (29.2)	0.25	528 (91.3)
Education level							
Others	187 (32.4)	77 (13.3)	1.00	177 (30.6)	87 (15.1)	1.00	264 (45.7)
University or above	203 (35.1)	111 (19.2)	0.11	212 (36.7)	102 (17.6)	0.90	314 (54.3)
Marital status							
Single	199 (34.4)	97 (16.8)	1.00	187 (32.4)	109 (18.9)	1.00	296 (51.2)
Have boyfriend(s)	141 (24.4)	67 (11.6)	0.90	149 (25.8)	59 (10.2)	0.05	208 (36.0)
Married	33 (5.7)	16 (2.8)	0.99	34 (5.9)	15 (2.6)	0.40	49 (8.5)
Others	17 (2.9)	8 (1.4)	0.94	19 (3.3)	6 (1.0)	0.20	25 (4.3)
Employment status							
Others	125 (21.6)	71 (12.3)	1.00	129 (22.3)	67 (11.6)	1.00	196 (33.9)
Full-time	265 (45.8)	117 (20.2)	0.17	260 (45.0)	122 (21.1)	0.59	382 (66.1)
Monthly income (yuan)							
0–2000	107 (18.5)	47 (8.1)	1.00	108 (18.7)	46 (8.0)	1.00	154 (26.6)
2001–4000	120 (20.8)	51 (8.8)	0.89	108 (18.7)	63 (10.9)	0.18	171 (29.6)
4001–5500	71 (12.3)	39 (6.7)	0.40	71 (12.3)	39 (6.7)	0.34	110 (19.0)
5501– 10 000	79 (13.7)	39 (6.7)	0.66	86 (14.9)	32 (5.5)	0.62	118 (20.4)
>10 000	13 (2.2)	12 (2.1)	0.09	16 (2.8)	9 (1.6)	0.54	25 (4.3)
Sexual orientation							
Gay	321 (55.5)	149 (25.8)	1.00	316 (54.7)	154 (26.6)	1.00	470 (81.3)
Bisexual	57 (9.9)	34 (5.9)	0.29	59 (10.2)	32 (5.5)	0.66	91 (15.7)
Others	12 (2.1)	5 (0.9)	0.84	14 (2.4)	3 (0.5)	0.20	17 (2.9)
Total	390 (67.5)	188(32.5)		389 (67.3)	189 (32.7)		100.0

*n* = number of subjects, *N* = number of total subjects, % = proportions.

In addition, the lifetime rate of IPV victimisation was 32.7% (189/578) and the lifetime rate of IPV perpetration was 32.5% (188/578). To be more specific (Table 2), among the five types of IPV, sexual violence victimisation (6.9%, 40/578) led more frequently to victim-only experiences, followed by monitoring behaviour victimisation (5.7%, 33/578). In terms of perpetrator-only experiences, people who perpetrated monitoring behaviour (5.9%, 34/578) and emotional violence (5.4%, 31/578) ranked first and second, respectively. Similarly, bidirectional violence was most often found in cases of emotional violence (11.8%, 68/578) and monitoring behaviour (9.3%, 54/578). The rate of bidirectional violence was higher than that of perpetrator-only and victim-only violence in cases of physical, monitoring and emotional violence, and the rate of victim-only violence in cases of sexual violence was higher than that of perpetrator-only or bidirectional violence.

**Table 2.** Descriptive statistics of lifetime IPV experience

Types of IPV *n* (%)	Physical	Sexual	Monitoring	Controlling	Emotional
Non-violence	511 (88.4)	497 (86.0)	457 (79.1)	507 (87.7)	448 (77.5)
Victim-only	17 (2.9)	40 (6.9)	33 (5.7)	27 (4.7)	31 (5.4)
Perpetrator-only	12 (2.1)	14 (2.4)	34 (5.9)	18 (3.1)	31 (5.4)
Bidirectional violence	38 (6.6)	27 (4.7)	54 (9.3)	26 (4.5)	68 (11.8)

*n* = number of subjects, % = proportions.

In this study, 36.0% (208/578) of the participants had significant depressive symptoms based on the CESD-10. In addition, 42.6% (246/578) were in poor general mental health based on the GHQ-12. In the past 3 months, 13.7% (79/578) reported suicidal ideation, 3.1% (18/578) had plans to commit suicide and 2.4% (14/578) had actually attempted suicide, resulting in a rate of suicidality 14.0% (81/578).

The mean scores for cognitive reappraisal and expressive suppression were 31.4 (s.d. = 5.7) and 16.0 (s.d. = 4.6), respectively. Cognitive reappraisal showed a negative association with the three adverse mental health outcomes adjusted for background variables (ORa = 0.98–0.92), whilst expressive suppression did not show a significant association with any of the three mental health outcomes.

### Associated factors of suicidality

In univariate analysis, age, ethnicity and marital status showed significant or marginally significant associations with suicidality, which were included in the adjusted multivariate logistic regression as controlled variables. Victim-only experiences of physical violence (ORa = 3.22, 95% CI = 1.11–9.32), victim-only experiences of sexual violence (ORa = 2.90, 95% CI = 1.39–6.05) and IPV victimisation (ORa = 1.90, 95%CI = 1.17–3.09) showed significant associations with suicidality in multivariate analysis. Similarly, adjusted logistic regression revealed that perpetrator-only experiences of monitoring behaviour (ORa = 2.93, 95% CI = 1.30–6.60), bidirectional experiences of monitoring behaviour (ORa = 3.01, 95% CI = 1.49–6.05) and IPV perpetration (ORa = 1.71, 95% CI = 1.05–2.79) showed positive associations with suicidality (Table 3).

**Table 3.** Adjusted logistic regression analysis of IPV experience and mental health outcomes

	Suicidality		Poor general mental health		Significant depressive symptoms	
	*p*	ORa (95% CI)^a^	*p*	ORa (95% CI)^b^	*p*	ORa (95% CI)^c^
Physical violence						
Non-violence		1.00		1.00		1.00
Victim-only	**0.03**	3.22 (1.11, 9.32)	0.18	1.98 (0.73, 5.39)	**0.05**	2.87 (1.02, 8.07)
Perpetrator-only	0.22	0.46 (0.14, 1.59)	0.35	0.53 (0.14, 2.03)	0.72	1.26 (0.35, 4.51)
Bidirectional violence	0.15	1.66 (0.83, 3.31)	0.17	1.60 (0.81, 3.16)	**0.03**	2.15 (1.06, 4.36)
Sexual violence						
Non-violence		1.00		1.00		1.00
Victim-only	**<0.001**	2.90 (1.39, 6.05)	**0.01**	2.53 (1.27, 5.02)	0.08	1.83 (0.94, 3.56)
Perpetrator-only	0.06	3.18 (0.95, 10.61)	0.83	1.12 (0.38, 3.32)	0.39	1.63 (0.54, 4.92)
Bidirectional violence	0.57	0.65 (0.15, 2.87)	0.63	1.22 (0.55, 2.72)	0.07	2.13 (0.93, 4.88)
Monitoring behaviour						
Non-violence		1.00		1.00		1.00
Victim-only	0.85	1.11 (0.37, 3.35)	0.30	1.47 (0.71, 3.03)	0.29	1.50 (0.71, 3.18)
Perpetrator-only	**0.01**	2.93 (1.30, 6.60)	**0.01**	2.89 (1.38, 6.07)	**<0.001**	4.45 (2.04, 9.68)
Bidirectional violence	**<0.001**	3.01 (1.49, 6.05)	**<0.001**	2.88 (1.58, 5.24)	**<0.001**	3.61 (1.95, 6.69)
Controlling behaviour						
Non-violence		1.00		1.00		1.00
Victim-only	0.05	2.46 (0.98, 6.15)	0.67	1.19 (0.54, 2.62)	0.86	0.93 (0.41, 2.12)
Perpetrator-only	0.81	0.83 (0.18, 3.79)	**0.04**	2.95 (1.07, 8.15)	0.07	2.50 (0.92, 6.77)
Bidirectional violence	0.34	1.65 (0.59, 4.61)	**0.07**	2.13 (0.94, 4.85)	**0.01**	3.41 (1.44, 8.12)
Emotional violence						
Non-violence		1.00		1.00		1.00
Victim-only	0.82	1.13 (0.41, 3.11)	**<0.001**	4.16 (1.80, 9.61)	**0.01**	2.97 (1.37, 6.42)
Perpetrator-only	0.55	1.36 (0.49, 3.73)	**0.02**	2.41 (1.13, 5.13)	**0.01**	2.92 (1.37, 6.25)
Bidirectional violence	0.64	1.19 (0.58, 2.44)	0.22	1.38 (0.82, 2.33)	**0.03**	1.86 (1.08, 3.21)
**Any IPV victimisation**	**0.01**	1.90 (1.17, 3.09)	**<0.001**	1.88 (1.32, 2.70)	**<0.001**	1.87 (1.29, 2.71)
**Any IPV perpetration**	**0.03**	1.71 (1.05, 2.79)	**<0.001**	1.74 (1.22, 2.50)	**<0.001**	2.30 (1.58, 3.36)
**Cognitive Reappraisal**	**<0.001**	0.92 (0.88, 0.96)	**<0.001**	0.89 (0.86, 0.92)	**<0.001**	0.87 (0.84, 0.91)
**Expressive Suppression**	0.91	1.00 (0.95, 1.06)	0.62	1.01 (0.97, 1.05)	0.64	1.01 (0.97, 1.05)

a Adjusted for Age; Ethnicity; Marital status.

b Adjusted for Marital status; Monthly income.

c Adjusted for Age; Ethnicity; Marital status; Monthly income.

ORa, adjusted odds ratio, *p* < 0.05 considered significant (in bold).

### Associated factors of poor general mental health

Adjusted for marital status and monthly income, IPV victimisation (ORa = 1.88, 95% CI = 1.32–2.70) and IPV perpetration (ORa = 1.74, 95% CI = 1.22–2.50) showed significant associations with poor general mental health. Other significant factors of poor general mental health included (Table 3): (1) victim-only experiences of sexual violence (ORa = 2.53, 95% CI = 1.27–5.02) and emotional violence (ORa = 4.16, 95% CI = 1.80–9.61); (2) perpetrator-only experiences of monitoring behaviour (ORa = 2.89, 95% CI = 1.38–6.07), controlling behaviour (ORa = 2.95, 95% CI = 1.07–8.15) and emotional violence (ORa = 2.41, 95% CI = 1.13–5.13); and 3) bidirectional experiences of monitoring behaviour (ORa = 2.88, 95% CI = 1.58–5.24) and controlling behaviour (ORa = 2.13, 95% CI = 0.94–4.85), after adjustment.

### Associated factors of significant depressive symptoms

After adjusting for age, ethnicity, marital status and monthly income, the following variables showed significant associations with significant depressive symptoms (Table 3): (1) victim-only experiences of physical violence (ORa = 2.87, 95% CI = 1.02–8.07) and emotional violence (ORa = 2.97, 95% CI = 1.37–6.42); (2) perpetrator-only experiences of monitoring behaviour (ORa = 4.45, 95% CI = 2.04–9.68) and emotional violence (ORa = 2.92, 95% CI = 1.37–6.25); (3) bidirectional experiences of physical violence (ORa = 2.15, 95% CI = 1.06–4.36), monitoring behaviour (ORa = 3.61, 95% CI = 1.95–6.69), controlling behaviour (ORa  = 3.41, 95% CI = 1.44–8.12) and emotional violence (ORa = 1.86, 95% CI = 1.08–3.21); and (4) IPV victimisation (ORa = 1.87, 95% CI = 1.29–2.71) and IPV perpetration (ORa = 2.30, 95% CI = 1.58–3.36).

### Interaction between ER and IPV on mental health problems

As shown in Table 4, significant correlations were seen (1) between one potential moderator (i.e., cognitive reappraisal) and the dependent variables (i.e., the three mental health outcomes); (2) between one potential moderator (i.e., cognitive reappraisal) and the independent variables (i.e., IPV victimisation and IPV perpetration); and (3) between the independent variables and the dependent variables. However, only the moderating effect of cognitive reappraisal was found in the association between IPV victimisation and poor general mental health (*B* = 0.07, *p* < 0.05; Table 5). This moderating effect indicated that a higher level of cognitive reappraisal mitigated the adverse effects of IPV experiences on general mental health more among non-victims than among victims.

**Table 4.** Correlations of study variables

	*n*/M	%/s.d.	1	2	3	4	5	6	7
1.Suicidality	81	14.0	1.00						
2.Significant depressive symptoms	208	36.0	0.28**	1.00					
3.Poor general mental health	309	53.5	0.25**	0.40**	1.00				
4.Any IPV victimisation	189	32.7	0.12**	0.15**	0.15**	1.00			
5.Any IPV perpetration	188	32.5	0.09*	0.17**	0.12**	0.61**	1.00		
6.Cognitive reappraisal	31.4	5.7	−0.17**	−0.35**	−0.29**	−0.11**	−0.09*	1.00	.
7.Expressive suppression	16.0	4.6	0.01	0.03	0.02	−0.03	−0.02	0.14**	1.00

*n,* number of subject; M,  mean; %, proportions; s.d.,  standard deviation.

* *p* < 0.05, **p <0.01.

**Table 5.** Final model for cognitive reappraisal and any IPV victimisation predicting poor mental health

Poor general mental health					
				95% CI	
	*B*	s.e.	ORa	Lower	Upper
Marital status					
Single			1.00		
Have boyfriend(s)	0.12	0.20	0.89	0.60	1.31
Married	0.59	0.35	1.80^#^	0.91	3.56
Others	0.28	0.47	0.75	0.30	1.90
Monthly income (yuan)					
0–2000			1.00		
2001–4000	0.04	0.24	0.96	0.60	1.52
4001–5500	0.19	0.28	0.83	0.48	1.43
5501– 10 000	0.60	0.28	0.55*	0.32	0.95
>10 000	0.40	0.48	0.67	0.26	1.72
**Any IPV victimisation**	0.61	0.19	1.84***	1.27	2.67
**Cognitive reappraisal**	0.11	0.02	0.89***	0.86	0.92
**Any IPV victimisation × Cognitive reappraisal**	0.07	0.04	1.08*	1.00	1.16
		Nagelkerke *R*^2^		*χ*^2^	
		0.17		79.97***	

# *p* < 0.1, * *p* < 0.05, **p < 0.01, *** *p* < 0.001.

## Discussion

This study investigated the lifetime prevalence of IPV and examined its association with mental health problems among MSM in China. The results confirmed the high prevalence of IPV in this population reported in previous studies (18.7%–51.0%; Dunkle *et al*., 2013; Davis *et al*., 2015; Ibragimov *et al*., 2017; Li and Zheng, 2017; Liu *et al*., 2018; Wang *et al*., 2018). We also found that the prevalence of bidirectional violence in cases of physical violence, monitoring behaviour and emotional violence was higher than that of perpetrator-only and victim-only violence. In other words, among these three types of violence, it is more likely to find a reverse cycle between the perpetrator and the victim, which may lead to more violent conditions.

In 2015, China passed its first law against domestic violence, which provides additional protection to minors, the elderly, people with disabilities, pregnant and lactating women, and seriously ill patients who suffer violence, but it ignores sexual minorities, such as MSM (Burki, 2017). Chinese MSM largely live in a heteronormative and homophobic environment (Zhang and Chu, 2005; Steward *et al*., 2013; Burki, 2017) and IPV in same-sex couples is relatively more invisible than in heterosexual couples. Due to the current absence of relevant laws and regulations against discrimination and domestic-violence, homosexual victims do not have access to adequate services and legal protection. This may further increase the effects of IPV on the mental health of Chinese MSM. The high prevalence of mental health problems found in this study (14.0% for suicidality, 53.5% for poor general mental health and 36.0% for significant depressive symptoms) is consistent with that of previous studies among MSM in China (10.6%–18.3% for suicidal ideation, 49.4% for poor general health and 26.8%–37.2% for depression; Mu *et al*., 2016; Li *et al*., 2016; Su *et al*., 2018; Wang *et al*., 2018; Zhu *et al*., 2018). In addition, this study discussed the effect of IPV, as a risk factor, and ER, as a potential moderator on the mental health of Chinese MSM.

Previous studies have focused mainly on the relationship between victims of violence and adverse psychological outcomes. Other roles, such as perpetrators of violence and bidirectional violence, and other forms of violence (e.g., emotional violence, controlling behaviour) have been less studied (Buller *et al*., 2014; Brown *et al*., 2016; Ibragimov *et al*., 2017). Therefore, one of the strengths of this study is that we investigated IPV in detail based on four roles and five types of violence and their specific effects on mental health. The results suggest that the roles of perpetrator and victim may have important implications for individual mental health status. For example, victims of physical and sexual violence were more likely to report suicidality than perpetrators, whilst the perpetrators of controlling and monitoring behaviours were more likely to have mental health problems than the victims. Previous studies of heterosexual couples found that different roles in violent experiences can have different effects on mental health problems (Anderson, 2004; Graham *et al*., 2012; Ulloa and Hammett, 2016). Overall, these studies have suggested that when designing interventions to improve the mental health status of MSM, attention should be paid to both the victims, especially victims of physical and sexual violence, and perpetrators, especially perpetrators of psychological violence (i.e. emotional violence, monitoring and controlling behaviour).

Although the concept of ER was first proposed and studied in western countries (Gross and John, 1998; Gross, 2002), it has been widely applied in eastern countries, including China, to study its effect on mental health in various populations. Hu *et al*.'s (2014) meta-analysis with a sample of 9454 Chinese participants and 8970 Western participants indicated that the correlation between expressive suppression and negative indicators of mental health (e.g. depression, anxiety and negative affect) was stronger in the Western cultural values category than in the Eastern cultural values category, whilst this cultural difference was not significant for cognitive reappraisal. Our results are consistent with those of previous studies. Many Eastern cultures such as China are deeply influenced by Confucianism. The Chinese collectivist culture emphasises social norms and group harmony and values individual restraint and the suppression of socially inappropriate emotions to maintain overall harmony. Therefore, expressive suppression may not have a negative effect on people who share this cultural value because it is consistent with their social value system (Hu *et al*., 2014). In addition, in response to the heteronormative culture prevalent in China, MSM may be used to hide their emotions, such as concealing their sexual orientation for social acceptance. Thus, expressive suppression may have a limited effect on the mental health of Chinese MSM (Sun *et al*., 2020).

The cultural context discussed above explains to some extent the results of this study. However, our results differ somewhat from our hypothesis because we found no significant association between expressive suppression and mental health. The buffering effect of cognitive reappraisal was found only in the association between IPV victimisation and poor general mental health, whilst higher cognitive reappraisal was significantly associated with better general mental health. ER is a complex psychological process influenced by many factors. Therefore, follow-up studies should examine relevant influencing factors, such as the cultural background, and further explore the mechanism of different ER strategies. Although GHQ scores decreased as the cognitive reappraisal scores increased in the victim group, this trend was not as significant as in the non-victim group. This indicates that cognitive reappraisal may be insufficient to mitigate the adverse effect of IPV victimisation on mental health in this population. Therefore, programs that seek to prevent mental health problems among MSM should integrate ER and other effective measures in their curriculum for IPV victims.

Studies of the different roles in IPV experiences and their effect on mental health among Chinese MSM have been limited. Therefore, this study is one of the first to examine the moderating effect of ER on the association between IPV and mental health. Moreover, the study sample included MSM from 15 cities across China, which makes the results more representative and generalisable. Despite these strengths, this study has several limitations. First, IPV is a taboo subject globally regardless of sexual orientation, gender and culture, for both perpetrators and victims. Therefore, the results of this study may be underestimated, thus affecting the identified effect of ER on IPV and mental health. Second, the ERQ is a widely used measure of ER developed in Western culture, and suppression is explicitly defined as one of its regulation modes (Gross and John, 2003). However, the suppression subscale includes only four items, which may not have enough power to differentiate participants in the Chinese context. Thus, the validity of this subscale did not allow us to explore the association of expressive suppression with IPV and ER.

## Conclusions

This study explored the prevalence of different perpetrator-victim roles in different types of IPV experiences, confirmed the adverse effect of IPV on mental health and tested its potential moderators among MSM in China. The effect of IPV on mental health differs due to different perpetrator-victim roles. Therefore, this study offers new insights into the development of prevention and intervention strategies. Targeted mental health inventions for MSM with IPV experiences should be tailored to their roles. In addition, cognitive reappraisal, as a protective factor for mental health, may not play a full role in victims of violence. Therefore, integrated interventions are needed for victims. Further research is warranted to investigate the effects of different perpetrator-victim roles in IPV experiences on mental health and identify potential positive moderators.

## Data Availability

The data sets used and analysed in the study are available from the corresponding author on reasonable request.

## References

[ref1] Anderson K (2004) Perpetrator or victim? Relationships between intimate partner violence and well-being. Journal of Marriage and Family 64, 851–863.

[ref2] Batchelder AW, Safren S, Mitchell AD, Ivardic I and O'cleirigh C (2017) Mental health in 2020 for men who have sex with men in the United States. Sexual Health 14, 59–71. 28055823 10.1071/SH16083 PMC5953431

[ref3] Brown MJ, Serovich JM and Kimberly JA (2016) Depressive symptoms, substance use and partner violence victimization associated with HIV disclosure among men who have sex with men. AIDS and Behavior 20, 184–192. 26122650 10.1007/s10461-015-1122-y PMC4696928

[ref4] Buller AM, Devries KM, Howard LM and Bacchus LJ (2014) Associations between intimate partner violence and health among men who have sex with men: a systematic review and meta-analysis. PLoS Medicine 11, e1001609. 24594975 10.1371/journal.pmed.1001609 PMC3942318

[ref5] Burki T (2017) Health and rights challenges for China's LGBT community. Lancet (London, England) 389, 1286. 10.1016/S0140-6736(17)30837-1 28379143

[ref6] Cutuli D (2014) Cognitive reappraisal and expressive suppression strategies role in the emotion regulation: an overview on their modulatory effects and neural correlates. Frontiers in Systems Neuroscience 8, 175–175. 25285072 10.3389/fnsys.2014.00175 PMC4168764

[ref7] Davis A, Best J, Wei C, Luo J, Van Der Pol B, Meyerson B, Dodge B, Aalsma M and Tucker J (2015) Intimate partner violence and correlates with risk behaviors and HIV/STI diagnoses among men who have sex with men and men who have sex with men and women in China: a hidden epidemic. Sexually Transmitted Disease 42, 387–392. 10.1097/OLQ.0000000000000302 PMC4520252 26222752

[ref8] Davis A, Kaighobadi F, Stephenson R, Rael C and Sandfort T (2016) Associations between alcohol use and intimate partner violence among men who have sex with men. LGBT Health 3, 400–406. 27906642 10.1089/lgbt.2016.0057 PMC5165665

[ref9] Devries KM, Mak JY, Bacchus LJ, Child JC, Falder G, Petzold M, Astbury J and Watts CH (2013) Intimate partner violence and incident depressive symptoms and suicide attempts: a systematic review of longitudinal studies. PLoS Medicine 10, e1001439. 23671407 10.1371/journal.pmed.1001439 PMC3646718

[ref10] Dunkle KL, Wong FY, Nehl EJ, Lin L, He N, Huang J and Zheng T (2013) Male-on-male intimate partner violence and sexual risk behaviors among money boys and other men who have sex with men in Shanghai, China. Sexually Transmitted Diseases 40, 362–365. 23588124 10.1097/OLQ.0b013e318283d2af PMC8080435

[ref11] Goldberg DP and Williams P (1988) A User's Guide to the General Health Questionnaire. Windsor: nferNelson.

[ref12] Goldberg DP, Oldehinkel T and Ormel J (1998) Why GHQ threshold varies from one place to another. Psychological Medicine 28, 915–921. 9723146 10.1017/s0033291798006874

[ref13] Graham K, Bernards S, Flynn A, Tremblay PF and Wells S (2012) Does the relationship between depression and intimate partner aggression vary by gender, victim-perpetrator role, and aggression severity? Violence and Victims 27, 730–743. 23155723 10.1891/0886-6708.27.5.730

[ref14] Gross JJ (2002) Emotion regulation: affective, cognitive, and social consequences. Psychophysiology 39, 281–291. 12212647 10.1017/s0048577201393198

[ref15] Gross JJ and John OP (1998) Mapping the domain of expressivity: multimethod evidence for a hierarchical model. Journal of Personality and Social Psychology 74, 170–191. 9457781 10.1037//0022-3514.74.1.170

[ref16] Gross JJ and John OP (2003) Individual differences in two emotion regulation processes: implications for affect, relationships, and well-being. Journal of Personality and Social Psychology 85, 348–362. 12916575 10.1037/0022-3514.85.2.348

[ref17] Hu T, Zhang D, Wang J, Mistry R, Ran G and Wang X (2014) Relation between emotion regulation and mental health: a meta-analysis review. Psychological Reports 114, 341–362. 24897894 10.2466/03.20.PR0.114k22w4

[ref18] Ibragimov U, Harnisch JA, Nehl EJ, He N, Zheng T, Ding Y and Wong FY (2017) Estimating self-reported sex practices, drug use, depression, and intimate partner violence among MSM in China: a comparison of three recruitment methods. AIDS Care 29, 125–131. 27367038 10.1080/09540121.2016.1201191 PMC8080274

[ref19] Kazan D, Calear AL and Batterham PJ (2016) The impact of intimate partner relationships on suicidal thoughts and behaviours: a systematic review. Journal of Affective Disorders 190, 585–598. 26583348 10.1016/j.jad.2015.11.003

[ref20] Kirwan M, Lanni DJ, Warnke A, Pickett SM and Parkhill MR (2019) Emotion regulation moderates the relationship between alcohol consumption and the perpetration of sexual aggression. Violence Against Women 25, 1053–1073. 30360699 10.1177/1077801218808396

[ref21] Lazarus RS and Alfert E (1964) Short-circuiting of threat by experimentally altering cognitive appraisal. Journal of Abnormal and Social Psychology 69, 195–205. 10.1037/h0044635 14213291

[ref22] Li D and Zheng L (2017) Intimate partner violence and controlling behavior among male same-sex relationships in China: relationship with ambivalent sexism. Journal of Interpersonal Violence. doi: 10.1177/0886260517724835. 29294885

[ref23] Li R, Cai Y, Wang Y, Sun Z, Zhu C, Tian Y, Jiang X and Gan F (2016) Psychosocial syndemic associated with increased suicidal ideation among men who have sex with men in Shanghai, China. Health Psychology 35, 148–156. 26462059 10.1037/hea0000265

[ref24] Liu Y, Zhang Y, Ning Z, Zheng H, Ding Y, Gao M, Wong FY and He N (2018) Intimate partner violence victimization and HIV infection among men who have sex with men in Shanghai, China. BioScience Trends 12, 142–148. 29760357 10.5582/bst.2018.01035

[ref25] Miller WC, Anton HA and Townson AF (2008) Measurement properties of the CESD scale among individuals with spinal cord injury. Spinal Cord 46, 287–292. 17909558 10.1038/sj.sc.3102127

[ref26] Mu H, Li Y, Liu L, Na J, Yu L, Bi X, An X, Gu Y, Zhou Y, Li S, Zhang R, Jiang C and Pan G (2016) Prevalence and risk factors for lifetime suicide ideation, plan and attempt in Chinese men who have sex with men. BMC Psychiatry 16, 117. 27129468 10.1186/s12888-016-0830-9 PMC4850688

[ref27] O‘Cleirigh C, Magidson JF, Skeer MR, Mayer KH and Safren SA (2015) Prevalence of psychiatric and substance abuse symptomatology among HIV-infected gay and bisexual men in HIV primary care. Psychosomatics 56, 470–478. 25656425 10.1016/j.psym.2014.08.004 PMC4339664

[ref28] Parker RD, Lohmus L, Valk A, Mangine C and Ruutel K (2015) Outcomes associated with anxiety and depression among men who have sex with men in Estonia. Journal of Affective Disorders 183, 205–209. 26025366 10.1016/j.jad.2015.05.014

[ref29] Radloff LS (1977) The CES-D scale: a self-report depression scale for research in the general population. Applied Psychological Measurement 1, 385–401.

[ref30] Shorey RC, Mcnulty JK, Moore TM and Stuart GL (2015) Emotion regulation moderates the association between proximal negative affect and intimate partner violence perpetration. Prevention Science 16, 873–880. 25995047 10.1007/s11121-015-0568-5

[ref31] Stephenson R and Finneran C (2013) The IPV-GBM scale: a New scale to measure intimate partner violence among gay and bisexual men. PLoS ONE 8, e62592. 23755098 10.1371/journal.pone.0062592 PMC3674004

[ref32] Stephenson R and Finneran C (2017) Receipt and perpetration of intimate partner violence and condomless anal intercourse among gay and bisexual men in Atlanta. AIDS and Behavior 21, 2253–2260. 28176169 10.1007/s10461-017-1709-6 PMC5534189

[ref33] Stephenson R, Hall CD, Williams W, Sato K and Finneran C (2013) Towards the development of an intimate partner violence screening tool for gay and bisexual men. Western Journal of Emergency Medicine 14, 390–400. 23997849 10.5811/westjem.3.2013.15597 PMC3756706

[ref34] Stephenson R, Freeland R and Finneran C (2016) Intimate partner violence and condom negotiation efficacy among gay and bisexual men in Atlanta. Sexual Health 13, 366–372. doi: 10.1071/SH15212 27120351

[ref35] Steward WT, Miège P and Choi KH (2013) Charting a moral life: the influence of stigma and filial duties on marital decisions among Chinese men who have sex with men. PLoS One 8, e71778. 23951245 10.1371/journal.pone.0071778 PMC3739721

[ref36] Su X, Zhou AN, Li J, Shi LE, Huan X, Yan H and Wei C (2018) Depression, loneliness, and sexual risk-taking among HIV-negative/unknown men who have sex with men in China. Archives of Sexual Behavior 47, 1959–1968. 29147806 10.1007/s10508-017-1061-y PMC5955768

[ref37] Sun S, Pachankis JE, Li X and Operario D (2020) Addressing minority stress and mental health among Men Who Have Sex with Men (MSM) in China. Current HIV/AIDS Reports 17, 35–62. 31950336 10.1007/s11904-019-00479-w PMC7050812

[ref38] Ulloa EC and Hammett JF (2016) The effect of gender and perpetrator–victim role on mental health outcomes and risk behaviors associated with intimate partner violence. Journal of Interpersonal Violence 31, 1184–1207. 25524265 10.1177/0886260514564163 PMC11293095

[ref39] Wang HY, Wang N, Chu ZX, Zhang J, Mao X, Geng WQ, Jiang YJ, Shang H and Xu JJ (2018) Intimate partner violence correlates with A higher HIV incidence among MSM: a 12-month prospective cohort study in Shenyang, China. Scientific Reports 8, 2879. 29440761 10.1038/s41598-018-21149-8 PMC5811488

[ref40] Wei D, Hou F, Hao C, Gu J, Dev R, Cao W, Peng L, Gilmour S, Wang K and Li J (2019) Prevalence of intimate partner violence and associated factors among men who have sex with men in China. Journal of Interpersonal Violence 0886260519889935. 10.1177/0886260519889935 31789088

[ref41] Zhang BC and Chu QS (2005) MSM And HIV/AIDS in China. Cell Research 15, 858–864. 16354560 10.1038/sj.cr.7290359 PMC1791010

[ref42] Zhang W, O‘Brien N, Forrest JI, Salters KA, Patterson TL, Montaner JS, Hogg RS and Lima VD (2012) Validating a shortened depression scale (10 item CES-D) among HIV-positive people in British Columbia, Canada. PLoS One 7, e40793. 22829885 10.1371/journal.pone.0040793 PMC3400644

[ref43] Zhu Y, Liu J, Chen Y, Zhang R and Qu B (2018) The relation between mental health, homosexual stigma, childhood abuse, community engagement, and unprotected anal intercourse among MSM in China. Scientific Reports 8, 3984. 29507341 10.1038/s41598-018-22403-9 PMC5838107

